# Increased Mast Cell Activation in Mongolian Gerbils Infected by Hepatitis E Virus

**DOI:** 10.3389/fmicb.2018.02226

**Published:** 2018-10-02

**Authors:** Tianlong Liu, Peng Xiao, Ruiwen Li, Ruiping She, Jijing Tian, Jingyuan Wang, Jingjing Mao, Jun Yin, Ruihan Shi

**Affiliations:** ^1^Laboratory of Veterinary Pathology and Public Health, College of Veterinary Medicine, China Agricultural University, Beijing, China; ^2^Sanford School of Medicine, University of South Dakota, Vermillion, SD, United States; ^3^College of Traditional Chinese Veterinary Medicine, Hebei Agricultural University, Dingzhou, China

**Keywords:** mongolian gerbils, hepatitis E virus, experimental infection, mast cell, activation

## Abstract

Recently, mechanism study of hepatitis E virus (HEV) infection has attracted an increasing attention because of the growing rate of the acute hepatitis caused by the virus over the world. As an important initiate in the inflammation, mast cells (MCs) play a critical role in maintaining a healthy physiology. However, the function of the MCs in the acute hepatitis caused by HEV is still unclear. In the present study, mongolian gerbils infected by HEV were used as an animal model to evaluate the role of MCs in the HEV infection. The positive ELISA and RT-PCR results showed the gerbils was successfully infected with HEV. The number of mast cell in the liver and the small intestine in the infected animals were growing higher significantly than the control group. In addition, higher expression of the tryptase and 5-HT in the liver and the intestine detected by immunohistochemical method and western blot also indicate the activation of MCs in the infection. These results suggest that MCs play an important role in the hepatitis E.

## Introduction

Hepatitis E virus (HEV) is a non-enveloped, positive sense single-stranded RNA virus that belongs to the family Hepeviridae (Smith et al., 2014), which is one of the main causes of acute hepatitis in humans and many other species (Martin-Latil et al., 2016; Donnelly et al., 2017). Although, hepatitis E is largely a self-limiting disease (Rathod and Tripathy, 2014, 2016), pregnant women and fetals are exceptionally susceptible to HEV infection (Nan et al., 2017; Verma et al., 2017). The mortality rate of pregnant women in the third trimester of pregnancy infected with HEV is as high as 25%, extremely easy to death of the mother and fetus, abortion, premature delivery or stillbirth (Mancinelli et al., 2017; Naru et al., 2017). The reasons for hepatitis E-related high maternal and fetal mortalities are unclear (Ahn et al., 2017). In last dozen years, extraepatic lesions caused by HEV have received increasing attention due to their association with neurological symptoms, acute pancreatitis, severe thrombocytopenia, myositis, and kidney diseases (Lee et al., 2009; Nan and Zhang, 2016). Unfortunately, the mechanism of HEV infection and transmission is still unclear. The most current studies focus on the HEV and antivirus immune signaling pathway including RIG-1, MAPK, NF-κB, and PI3K (Zafrullah et al., 1997; Korkaya et al., 2001; Zhou et al., 2014; He et al., 2016; Tian et al., 2017; Xu et al., 2017). More studies are required to fully understand the mechanism of HEV infection.

Mast cells (MCs) not only play important role in allergic responses but also are employed as a initiator and regulator cells in both innate and adaptive immune responses (da Silva et al., 2014). It is well known the widely distribution of MCs in the body, especially, at the skin and airways recognized as host environmental interfaces, where they can be found around nerves and blood vessels (Wang et al., 2012; Ronsard et al., 2017b). Therefore, they can releasing many active mediators such as cytokines, chemokines, and lipid mediators for the purpose of acting as important sentinels for the immune system and control effective innate responses against invading pathogens (Ronsard et al., 2014; Wouters et al., 2016). Unlike the recognized contributions of MCs to host defense against bacteria, the function of MCs in antiviral immunity has not been well understand (Zizzi et al., 2011; Graham et al., 2013; Swindle et al., 2015; Ronsard et al., 2017a). In recent researches, several viruses, including dengue virus and infectious bursal disease virus, have been suspected can activate MCs *in vitro* through TLR3, TLR7, and possibly other mechanisms (Wang et al., 2008, 2009; Chu et al., 2015, 2017). However, the *in vivo* contribution of MCs to host defense in viral infections is need more investigation.

In recent, researches of viral hepatitis, liver cirrhosis, liver fibrosis and other bile duct disease have shown that mast cell usually increases as the disease development, suggest MCs play important role in the liver disorders (Heine et al., 1978; O’Keeffe et al., 2002; Franceschini et al., 2007). In systemic mast cell activation syndrome patients associated with elevated cholesterol levels also showed high transaminase and bilirubin levels. This finding suggested that mast cell activation involving in the abnormal of the liver function (Bardadin and Scheuer, 1986; Doria et al., 2006). MCs has been considered as one indicators of the acute hepatitis. However, special roles of MCs play in the liver disease remain unknown. Moreover, very few information were reported on the activity of MCs in the hepatitis E.

In the present study, we examined the MCs activation in Mongolian gerbil model of HEV. Mongolian gerbil was successfully infected by genotype 4 strain of HEV via the intraperitoneal route. Using toluidine blue staining and immunohistochemical staining method, the distribution and release of mediators such as the tryptase and 5-HT of MCs in the liver and the intestine were investigated. Histopathological changes of major organs like liver, kidney, spleen, and brain also were examined in the study.

## Results

### Clinical Evaluation and Detection of HEV

**FIGURE 1:**
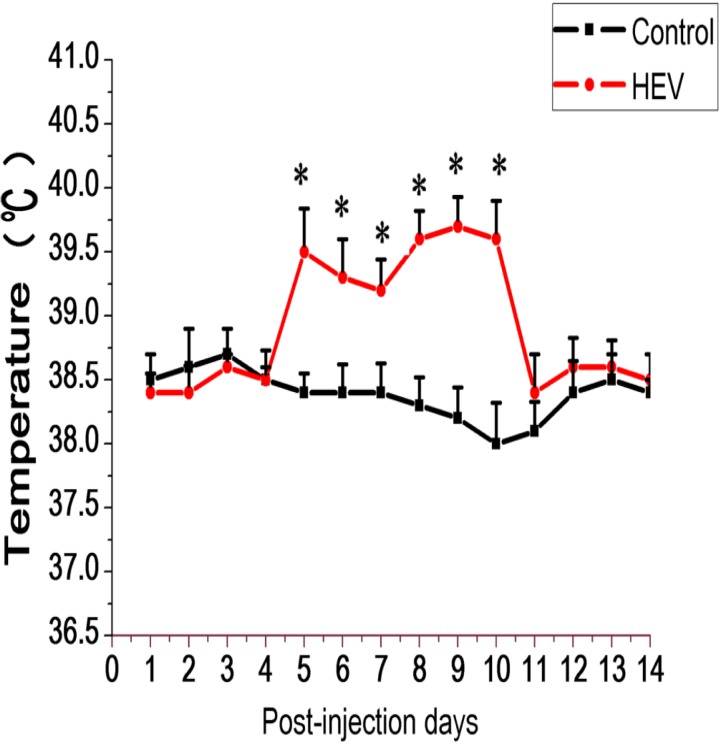
Schematic diagram body temperature of Gerbils inoculated with HEV recovering pig liver and negative pig liver. In the inoculated group, body temperature rose at 5–11 dpi. The normal body temperature of gerbils is between 38.1 and 38.4°C (^∗^Denotes statistical significance for the comparison of control, ^∗^*p* < 0.05).

For body temperature detection, the temperature of experiment group was observed to rise from 5 to 11 dpi, up to the 39.7°C, obviously higher than that of control group (**Figure 1**). The clinical manifestations in patients infected with HEV include fever, malaise, nausea, vomition, anorexia, abdominal distension, and diarrhea, at the same time, liver slightly enlargement, tenderness and percussion pain, urine color deepened. In general, those symptoms will last for several days, an average of 10 day. But for animals infected with HEV, it is not clear that whether fever would be observed. In the study, the body temperature of Mongolian gerbils inoculated with HEV was recorded daily, and the results showed that the gerbils had a fever in the early infection stage, but lasted only for a week. The most obvious increase is on the 9–10 dpi (the increase of about 1.5°C). This result indicate that animals infected with HEV can also present clinical symptoms such as fever, but due to the short duration and spontaneous recovery, it is often overlooked.

**Table 1** shows the results of positive tissue number for HEV RNA RT-PCR detection at different time points. After inoculation, all liver tissues (5/5) of the gerbils in experimental group at 14, 28, and 56 dpi were positive for RT-PCR detection. At 14 and 21 dpi, positive samples of the small intestine were 3/5 and 5/5. In the feces, 11/15 and 10/10 positive sample were detected at 14 and 28 dpi. The liver, serum, and feces of the control group gave negative results though the whole experiment (**Table 1**). The ELISA assay results are listed in **Table 2**. At 14, 28, and 56 dpi, 2/15, 6/10, and 3/5 positive serum of anti-HEV IgG were detected by ELISA, but no positive was observed in the control group during the experiment.

**Table 1:** HEV RNA detection of gerbils inoculated with HEV.

Group	Samples^a^	HEV RNA positive rate on weeks post inoculation			
		7 dpi	14 dpi	28 dpi	56 dpi
Experiment group	Feces	0/20	11/15	10/10	0/5
	Serum	12/20	15/15	10/10	0/5
	Liver	4/5	5/5	5/5	5/5
	Small intestine	0/5	3/5	5/5	0/5
	Kidney	0/5	0/5	0/5	0/5
	Spleen	0/5	0/5	0/5	0/5
Control group	Liver	0/5	0/5	0/5	0/5
	Serum	0/20	0/15	0/10	0/5
	Feces	0/20	0/15	0/10	0/5

*^a^For the experiment group, gerbils were necropsied and samples weekly, only liver, sera, and feces were tested for control group.*

**Table 2:** Anti-HEV IgG Detection of Gerbils Inoculated with HEV.

DPI	0 dpi	7 dpi	14 dpi	28 dpi	56 dpi
Control group	0/20	0/20	0/15	0/10	0/5
Experiment group	0/20	20/20	12/15	6/10	3/5

**FIGURE 2:**
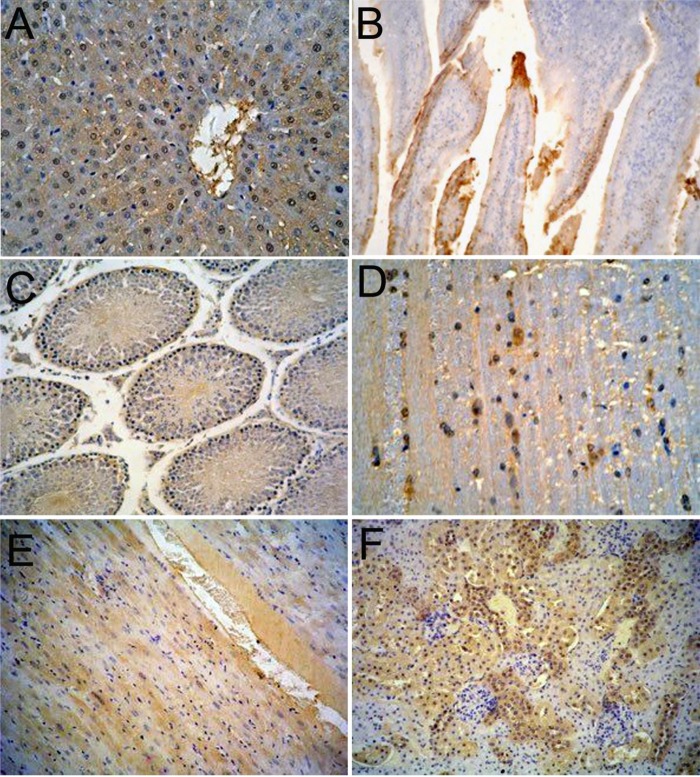
Immunohistochemical analysis of Mongolian gerbils’ tissues. HEV ORF2 positive signals expression scattered in the cytoplasm of hepatic cells **(A)**, the cytoplasm of mucosal epithelial cells **(B)**, the cytoplasm of spermatogenic cells **(C)**, the cytoplasm of neurons **(D)**, the cytoplasm of muscle fiber **(E),** and in renal tubular epithelial cells in the cytoplasm **(F)**. (Magnification: 20×).

In order to observe the distribution and expression of HEV ORF2 in major organs, immunohistochemical staining was performed. Positive signals for HEV-ORF2 proteins were represented by a brown or yellow granular mass. There were strong positive signals for ORF2 were observed in the major tissues of the experimental group (**Figure 2**). On the contrary, no positive signals for the HEV ORF2 antigens were detected in the major tissues in the control group (**Supplementary Figure S1**). Immunohistochemistry showed that HEV ORF2 positive signals were detected in the liver hepatocyte around the portal area (**Figure 2A**), the epithelial cell of the small intestine (**Figure 2B**), seminiferous epithelium cell of the testis (**Figure 2C**), nerve cell of the brain (**Figure 2D**), myocardial cell of the heart (**Figure 2E**), and the renal tubular epithelial cell of the kidney (**Figure 2F**). The mainly type of positive feature was cytoplasmic diffusive, and the positive signals in later stage infection were more than in the early stage.

### Activation Detection of Mast Cell

**FIGURE 3:**
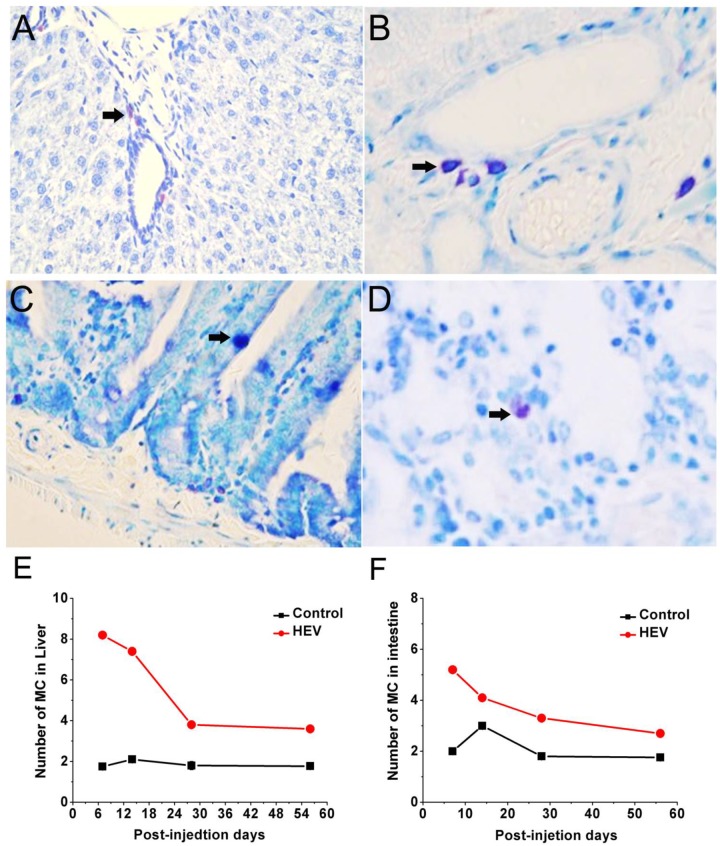
Toluidine blue staining for mast cells (black arrow) mainly concentrate in periportal vessels of the liver (**A,B**, magnification: 40×), in the muscle layer and lamina propria of small intestine (**C,D**, magnification: 40×). The quantity of mast cell in the livers **(E)** and the small intestine **(F)** of gerbils infected with HEV.

As shown in **Figure 3**, MCs in the liver and the small intestine were detected by toluidine blue staining and appeared in dark purple. For experiment group, MCs mainly concentrate in periportal vessels of the liver (**Figures 3A,B**) or in the muscle layer and lamina propria of small intestine (**Figures 3C,D**). Compared with the control group, number of MCs in the liver and the small intestine both increase significantly in the early infection stage and got the maximum on 7 dpi then phased down (**Figures 3E,F**).

**FIGURE 4:**
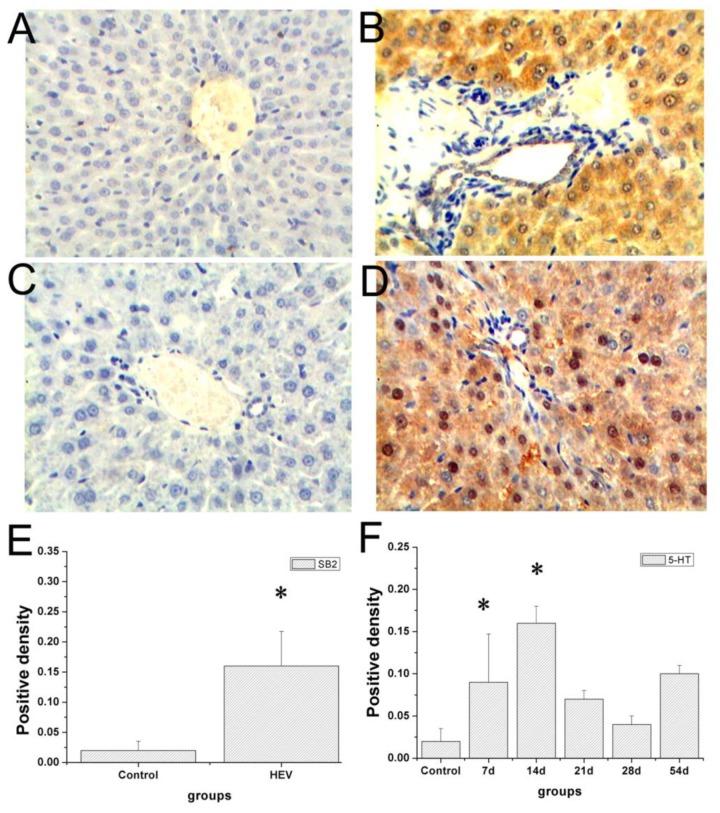
Immunohistochemical analysis of tryptase and 5-HT in the livers. No positive signals were observed in the control group **(A,C)**. Positive signals of tryptase (yellow) expression scattered in the cytoplasm of hepatic cells **(B)**, positive signals of 5-HT (brown) in the cytoplasm of hepatic cells **(D)** (Magnification: 20×). The density of tryptase **(E)** and 5-HT **(F)** positive signals in the livers of gerbils infected with HEV (^∗^Denotes statistical significance for the comparison of control, ^∗^*p* < 0.05).

**FIGURE 5:**
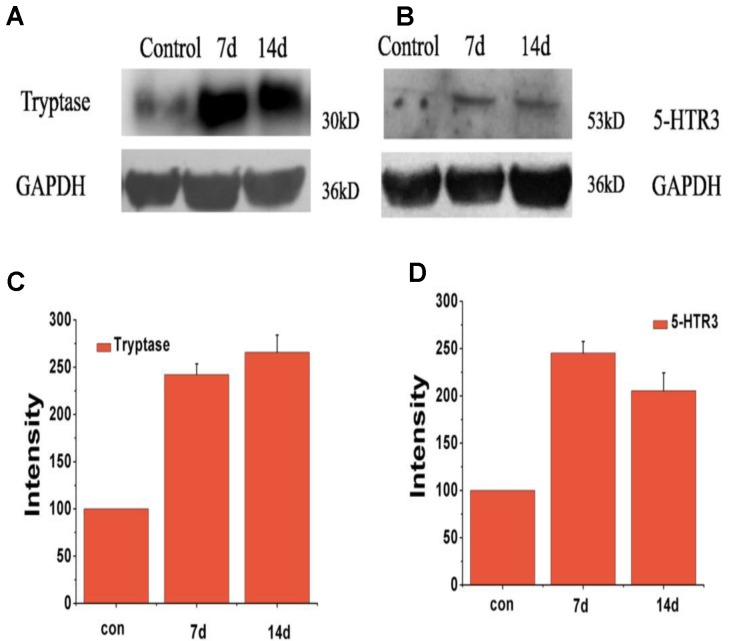
Expression level of protein of tryptase and 5-HTR3 in liver tissue of different groups at 7 and 14 dpi. **(A)** Western blot analysis of tryptase, **(B)** western blot analysis of 5-HTR3. **(C)** Intensity of expression of tryptase, **(D)** intensity of expression of 5-HTR3 (^∗^Denotes statistical significance for the comparison of control, ^∗^*p* < 0.05).

Immunohistochemistry staining images of the tryptase and 5-hydroxytryptamine in the liver were showed in **Figure 4**. No positive signals for tryptase and 5-hydroxytryptamine were detected in the liver in the control group (**Figures 4A,C**). Positive signals for tryptase appeared in yellow were observed in the cytoplasm of hepatocyte around the portal area (**Figure 4B**). Positive signals for 5-hydroxytryptamine appeared in brown were observed in the cytoplasm and cell nucleus of hepatocyte (**Figure 4D**). In the early infection stage, the positive density of tryptase and 5-hydroxytryptamine of experiment group was significantly higher than that of the control group (**Figures 4E,F**). The protein expression levels of tryptase and 5-hydroxytryptamine were detected by western blotting. **Figure 5** shows the expression of tryptase and 5-hydroxytryptamine were significant higher in HEV RNA-positive liver tissues than in the control group. These results suggested the increased MCs activation and MCs play an important role in the inflammation happen in the liver and intestine.

### Histopathological Observation

**FIGURE 6:**
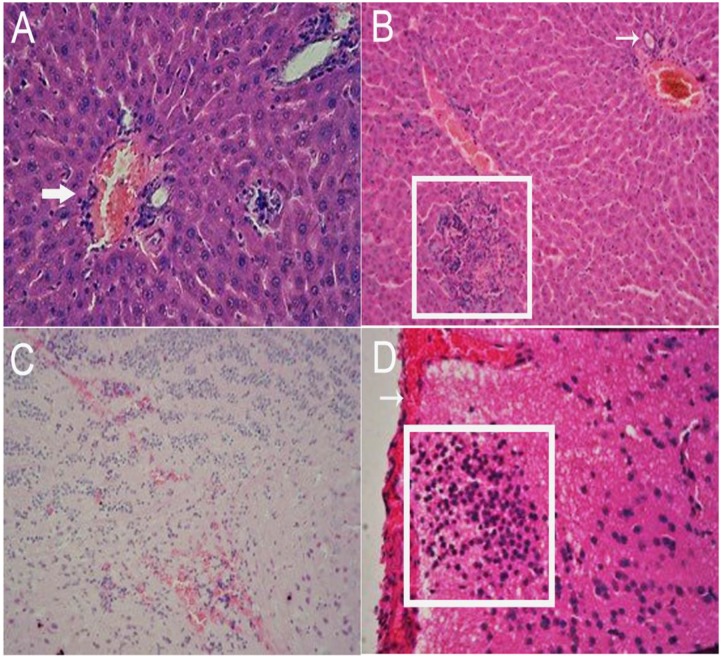
Histopathological analysis of liver and brain tissue section stained with hematoxylin and eosin (HE). In the liver, there are sinus congestion and central venous congestion (arrow), degeneration of hepatocyte granules (**A**, 40×), bile duct epithelial hyperplasia (arrow), hepatocyte necrosis (pane), and lymphocytic infiltrations were observed in Kiernan’s space (**B**, 20×). Focal hemorrhage (**C**, 20×), meningeal blood vessels dilation (arrow), glial nodule formation (pane) (**D**, 40×) can be observed in the brain.

**FIGURE 7:**
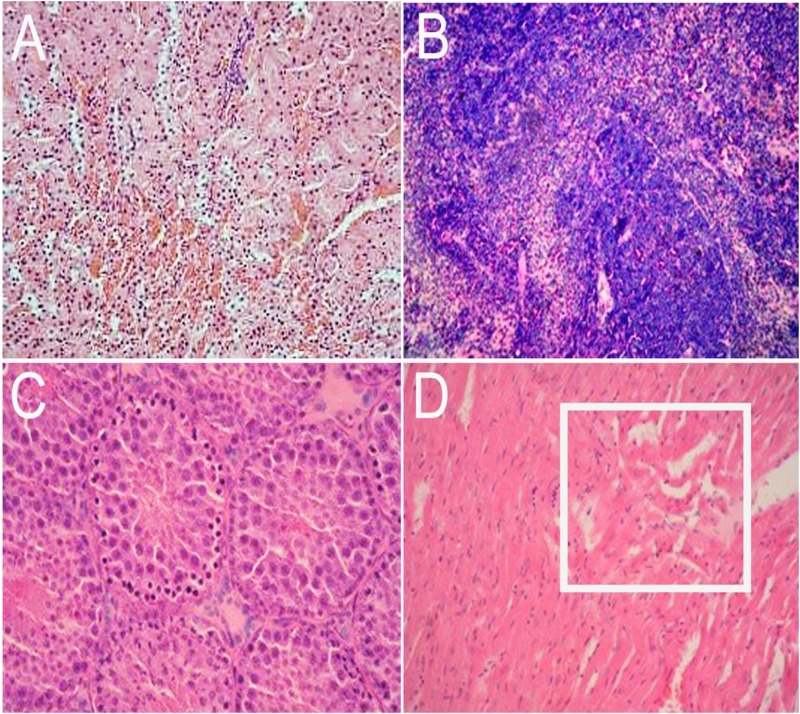
Histopathological analysis of kidney, spleen, testis, and heart tissue section stained with hematoxylin and eosin (HE). In the kidney, there are renal tubular epithelial cell degeneration and interstitial vascular congestion (**A**, 20×). There are some mild bleeding in the white pulp of spleen (**B**, 40×). The epithelial of spermatogenic cells arrange loosely and the nucleus is fixed in testis (**C**, 20×) and myocardial fiber focal necrosis (pane) in the heart (**D**, 20×).

Inoculated gerbils show pathological signs of HEV infection from the 14 DPI compared with the control group. In the liver, there are sinus congestion and central venous congestion, degeneration of hepatocyte granules (**Figure 6A**), bile duct epithelial hyperplasia, hepatocyte necrosis, and lymphocytic infiltrations were observed in Kiernan’s space (**Figure 6B**), there is also proliferation of fibrous connective tissue in portal tracts (**Supplementary Figure S2**) and hepatic sinusoidal reticular fiber turn into collagen in liver. Focal hemorrhage (**Figure 6C**), meningeal blood vessels dilation, glial nodule formation (**Figure 6D**). In the kidney, there are renal tubular epithelial cell degeneration and interstitial vascular congestion (**Figure 7A**). There is some mild bleeding in the white pulp of spleen (**Figure 7B**). The epithelial of spermatogenic cells arrange loosely and the fixed nucleus (**Figure 7C**) and myocardial fiber focal necrosis in the heart (**Figure 7D**) were observed. Meanwhile, the damage in later stage infection was more serious than in the early stage.

## Discussion

Because of the shortage of practical animal model and effective cell culture system limited the research of HEV (Huang et al., 2004), there is big challenge for effective HEV vaccine available (Dalton et al., 2008). The non-human primates and swine have been used as infection models effectively, but the cost limit the application of these animal models (Lee et al., 2009). Previously, we reported Mongolian gerbil can be used as an animal model for HEV, because of that Mongolian gerbil is sensitive to HEV and lower rates of spontaneous hepatitis (Soomro et al., 2016, 2017). In the present study, Mongolian gerbil hepatitis E infection model was successfully obtained according to the intraperitoneal route.

Mast cell can constantly monitor bacterial invasion as “sentinels,” and give rise to the immune response against pathogens (Schmerse et al., 2014). Mast cell can release different mediators and cytokines for different stimuli, as a result, it plays an important role in the initiation and prognosis of inflammatory response. MCs are recognized as a critical moderator in maintaining a healthy physiology and participating in innate and adaptive immunity. Mast cells are also involved in the inflammatory process, recruiting different white blood cells in injured sites and allergic diseases (Weller et al., 2011). Several studies have shown that MCs are associated with antiviral defense mechanisms and pathogenesis of viral diseases. In most cases, MCs are beneficial to the host’s defense response, but in some cases, it can also be damaging to the host. For example, MCs could promote lung injury caused by H5N1 influenza virus infection by releasing pro-inflammatory agents (Hu et al., 2012). MCs are also involved in host defense at herpes simplex virus-infected sites through tumor necrosis factor-α (TNF-α) and interleukin-6 (IL-6) production, which are induced by keratinocyte-derived IL-33 (Aoki et al., 2013). In the present study, the number of MCs and stimulated release of mediators such as tryptase and 5-hydroxytryptamine in liver and intestine were examined by different methods. Increased number of MCs and higher expression of tryptase and 5-hydroxytryptamine indicate that activation of MCs maybe involving in the HEV pathogenesis.

Tryptase is a neutral serine protease and is the most abundant mediator stored in mast cell granules. The release of tryptase from the secretory granules is a characteristic feature of mast cell degranulation. Mast cell tryptase has an important role in inflammation and serves as a marker of mast cell activation (Payne and Kam, 2004; Wang et al., 2016). The tryptase initiated signaling cascade supporting the tissue repair and sometimes fibrosis proliferative response has been elucidated in fibroblasts (Frungieri et al., 2005; Duchesne et al., 2011). 5-hydroxytryptamine (serotonin) is a monoamine neurotransmitter synthesized in serotonergic neurons in the central nervous system (CNS) and enterochromaffin cells in the gastrointestinal tract of animals including humans (Gruba et al., 2014). 5-HT is present in murine mucosal MCs and some authors reported that human MCs may also contain serotonin (Kritas et al., 2014). In our study, tryptase and 5-HT were used to evaluate the activation of MCs. The higher expression of these two indexes indicated that MCs cells activation during the HEV infection.

The main pathological damage in liver tissues of patients infected with HEV is portal inflammation, there are a large number of lymphocytes and kupffer cells infiltration (Wu et al., 2017). Usually, accompanied with cholangiole cholestasis, liver cell degeneration. For acute severe cases, liver cell diffuse necrosis, a mass of infiltration of lymphocytes, especially in portal tracts. For subacute severe cases, the structure of hepatic lobule is not clear, there are multiple focal necrosis among the normal tissues. In the study, we observed the histopathological lesion of all tissues of Mongolian gerbils infected with HEV. And the application of Mallory trichrome staining suggested that the liver fibrosis appeared in the later stage of infection.

## Conclusion

The present study demonstrated that Mongolian gerbils as an ideal animal model of HEV infection could be used in the mechanism study. In addition, the study provides a new idea for the further study of HEV pathogenesis, which enhanced MCs number and release of proteases and 5-HT to increased local vascular permeability and recruitment of other participants in the mongolian gerbils infected by HEV. Activation of mast cell may play an important role in the HEV infection, but the specific mechanism need further study as well. Moreover, it is urgent need to establish “MC knock-in animal” to evaluate the contribution of MCs in HEV infections.

## Materials and Methods

### Inoculum

SwHB-3 (HEV 4) recovered from a swine liver sample collected from Hebei province, China, used in the experiment. The part of the sequence of swHB-3 has been determined and deposited in Gene Bank (GU553094). In details, liver homogenates (10%, w/v, suspension) were filtered (0.22 mm filter unit, Carrigtwohill, Co., Cork, Ireland) and the PID50 (50% swine infectious doses per ml of inoculum) was determined as previously described (Meng et al., 1998).

### Animals and Treatment

Forty male Mongolian gerbils (Meriones unguiculatus) of clean grade were purchased from the Department of Experimental Animal Sciences of Capital Medical University (Beijing, China). All animals used in the experiment were confirmed negative for HEV antibodies examination by an ELISA assay. All treatments were performed following an acclimation period of 7 days after their arrival at the laboratory to avoid stress. Animals were randomly divided into two groups (the experimental group and control group). Each gerbil in the experimental group was inoculated via the intraperitoneal route with a 0.1 ml inoculum that had an infectious titer of 10^4.5^ PID50. Animal in the control group was inoculated with the equal volume of RT-PCR negative liver homogenates as negative controls. Temperature was measured daily during 14 days post injection (dpi). This study was carried out in accordance with the recommendations the Regulations of Experimental Animals of Beijing Authority. The protocol was approved by the Animal Ethics Committee of the China Agriculture University.

### Specimen Collection

Fecal samples and serum samples were collected weekly, then the samples were stored at −80°C. Body temperature of gerbils were measured daily. Five gerbils from each group were necropsied at 7, 14, 28, and 56 dpi, the heart, liver, spleen, lung, kidney, stomach, testis, small intestine, adrenal gland, and brain were collected at an immediate necropsy. Fresh frozen tissue samples were stored at −80°C for HEV RNA detection. Additional samples were fixed immediately in 2.5% (v/v) glutaraldehyde-polyoxymethylene solution for 72 h for histological studies and immunohistochemical staining. All samples stored at −80°C. Effective measures have been performed to avoid HEV cross contamination during necropsy sampling as previously described (de Deus et al., 2008).

### RT-PCR to Detect HEV RNA in Inoculated Gerbils

Viral RNA was extracted from the inoculated gerbils samples such as serum, feces, liver, spleen, kidney, and small intestine. The detailed procedure of RT-PCR is listed in the **Supplementary Materials**. Then nested RT-PCR was performed using two sets of ORF2-specific primers designed for detecting genetically divergent strains of HEV: HE164F1 (5′-GCR GTG GTT TCT GGG GTG AC-3′) and HE164R1 (5′-CTG GGM YTG GTC DCG CCA AG-3′) were used as out primers, and HE137F2 (5′-GYT GAT TCT CAG CCC TTC GC-3′) and HE137R2 (5′-GMY TGG TCD CGC CAA GHG GA-3′) used as internal primers (Tam et al., 1991). Detailed information of transcription system for RNA and amplifying system for HEV RNA RT-PCR are listed in **Supplementary Tables S1**, **S2**. Amplification conditions for both round of PCR were: 94°C for 3 min; 30 cycles of 94°C for 30 s, 58°C for 35 s, and 72°C for 30 s; 72°C for 7 min (Yang et al., 2018). Negative (pure water) and positive (HEV RNA) controls were designed in the RT-PCR assay.

### Histopathological Examination

The fixed tissues were dehydrated using increasing concentrations of ethanol and embedded in paraffin according to standard laboratory procedures, and 4 μm sections were prepared then stained with hematoxylin and eosin (HE) for histological evaluation (Yang et al., 2015). The unstained sections were used for immunohistochemistry, Mallory’s trichrome, and toluidine blue staining for antigen detection, tryptase and 5-hydroxytryptamine, fibrosis and MCs observations in tissues. The positive MCs stained by toluidine blue were observed as purplish red granular mass (Joseph et al., 2018). Multiple views (five per section of each organ) were used to count the number of positive cells per view. The average of each groups were calculated. All tissue sections were examined with an Olympus BH-2 microscope (Olympus Optical Co., Ltd., Beijing, China).

### Immunohistochemistry

Tissue sections were prepared as previously described in this section, were deparaffinating, hydrated, water-bath heated for antigen retrieval and blocked with the addition of 3% hydrogen peroxide for immunohistochemistry. Afterward sections were incubated overnight with primary antibody monoclonal mouse anti-HEV ORF2, tryptase and 5-hydroxytryptamine antibody (1:200 dilution; Beijing Protein Institute, Beijing, China). Immunohistochemical staining was performed following the instructions that were included in the Histostain^TM^-Plus kit (ZSGB-BIO, Beijing, China). 3,3′-Diaminobenzidine tetrahydrochloride (DAB; ZSGB-BIO, Beijing, China) was applied for 5 min to visualize the antigen–antibody compound, Gill’s hematoxylin was applied as the background stain (Yang et al., 2018). The slides were observed under light microscope (Olympus Optical Co., Ltd., Beijing, China) and positive signals for HEV-ORF2 proteins were represented by a brown or yellow granular mass (Soomro et al., 2016). The positive staining intensity of ORF2, tryptase, and 5-hydroxytryptamine were measured as the ratio of the stained area to the total field assessed. Multiple views (three fields per section and five sections per animal) were randomly selected and analyzed.

### ELISA for HEV Antibody and Antigen Detection

Serum samples were tested for the presence of IgG anti-HEV, using a commercial ELISA kit (Wan-tai Biological Pharmacy Co., Beijing, China) following the manufacturer’s instructions. The absorbance was determined at 450 nm (Multiscan Titer tek MCC). According to the instruction of Beijing Wantai Biological Pharmacy Enterprise Co., Beijing, China, cut off value is the half of the total of positive control OD value plus negative control OD value. Positive is identified if the test OD < cut off OD.

### Western Blotting Analysis

The protein expression levels of tryptase and 5-hydroxytryptamine receptor 3 were detected by western blot analysis. The tryptase antibody was obtained from Beyotime Biotechnolgoy Co., Ltd. (China). The 5-hydroxytryptamine receptor 3 antibodies were obtained from Biosynthesis Biotechnolgoy Co., Ltd. (China). The detailed procedure of WB analysis is listed in the **Supplementary Materials**.

### Statistical Analysis

Results were expressed as mean ± standard deviation (SD). Multigroup comparisons of the means were carried out by one-way analysis of variance (ANOVA) test using SPSS 16.0 (SPSS Inc., Chicago, IL, United States). The statistical significance for all tests was set at *p* < 0.05. All graphs were generated with Graph Pad Prism 5.0 (GraphPad Software, La Jolla, CA, United States).

## Author Contributions

TL, PX, JT, RL, and RPS are responsible for the research design and concept. PX, JW, RL, JY, RHS, and JM performed the experiments and laboratory work. TL, JT, and PX analyzed the data. TL, JT, and RPS wrote the manuscript. All authors have read, commented on, and approved the final article.

## Conflict of Interest Statement

The authors declare that the research was conducted in the absence of any commercial or financial relationships that could be construed as a potential conflict of interest.
