# Supplementary material for: Increased Mast Cell Activation in Mongolian Gerbils Infected by Hepatitis E Virus
Source: Front Microbiol. 2018 Oct 2;9:2226. doi: 10.3389/fmicb.2018.02226 (PMC6175998; doi:10.3389/fmicb.2018.02226)
Supplement: Supplementary file 1 [file Presentation_1.pdf]

Supplement materials

Increased Mast Cell Activation in Mongolian Gerbils Infected by Hepatitis E Virus

Tianlong Liu,[†‡] Peng Xiao, [⁑‡] Ruiwen Li,[‖ ‡] Ruiping She[†*], Jijing Tian,[†] Jingyuan Wang,[†] Jingjing Mao,[†] Jun Yin,[†] Ruihan, Shi[†]

† Laboratory of Veterinary Pathology and Public Health, College of Veterinary Medicine, China Agricultural University, No.2 West Road Yuanmingyuan, Beijing 100193, P.R. China

⁑ Sanford School of Medicine, university of South Dakota, vermillion, SD, 57069, United State

‖ College of Traditional Veterinary Medicine, Hebei Agricultural University, Dingzhou, China

E-mail addresses: sheruiping@126.com

‡ These authors contributed equally to this work

Key words: Mongolian Gerbils, hepatitis E virus, experimental infection, mast cell, activation

Materials and Methods

Procedure of RT-PCR to Detect HEV RNA in Inoculated Gerbils

Briefly, the samples (100mg of tissue) were disrupted with liquid nitrogen (with the exception of the serum samples). RNA was extracted using the Ultrapure RNA kit and the RNA pure Virus kit (CWBIO, Beijing, China) following the manufacturer’s instructions. The RNA was stored at −86 ◦C.

Western blotting analysis

Briefly, a small amount of Mongolian gerbil livers from each group were homogenized in lysis buffer containing 7M urea, 2M thiourea, 4% Chaps,1% DTT, 400 mM Tris base, and 1mM PMSF. After centrifugation at 12,000 rpm for 20 min at 4 ◦C, the supernatant was collected and used as tissue lysate. Protein concentrations were determined by Nanodrop 2000 spectrophotometer (Thermo, USA). Equal amounts of protein from each sample were boiled in SDS sample buffer for 10 min. The samples were separated by SDS-PAGE under reducing conditions and electro blotted onto polyvinylidene fluoride (PVDF) membranes. The PVDF membranes were then blocked by 5% skim milk for 1h and incubated with the primary monoclonal antibody (1:300 diluted) overnight at 4 ◦C. After washing, the membranes were incubated with a secondary antibody, horseradish peroxidase-conjugated goat anti-mouse IgG, at 37 ◦C for 1h. The conjugated substrate was detected with an enhanced chemilumine scence detection kit (CWBIO, China) and exposed to X-ray film. Equal protein loading was confirmed by staining with GAPDH antibody (Wuhan Boster BioEngineering Limited Company, China). Densitometric analysis of the Western blots was performed using Image Lab™ software (Quantity ONE7.0).

Table S1 Transcription system for RNA

Volume of agents(μL)	
dNTP mix	4
5xRT Buffer	4
Primer Mix	2
DTT	2
Rase Free water	0.5
HiFi-MMLV	1
RNA template	6.5
Total	20

Table S2 Amplifying system for HEV RNA RT-PCR

Agents	Volume（μL）
template	2.5
Primer1	0.5
Primer2	0.5
DdH_2O	6.5
PCR MIX	10
Total	20

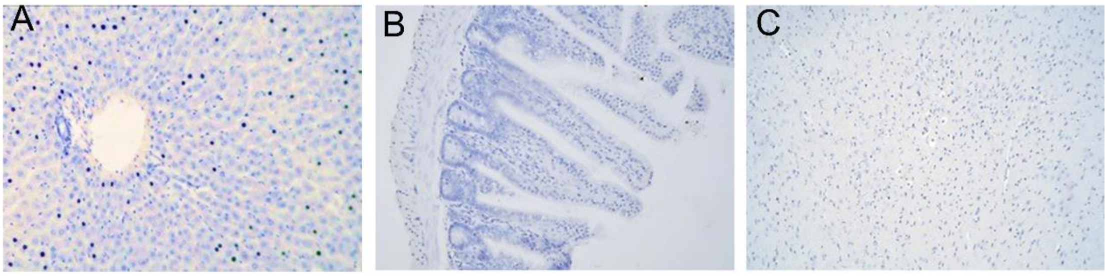

Figure S1 Immunohistochemical analysis of OFR 2 in the control group tissues.No positive signals for the HEV ORF2 antigens were detected in the liver (A), intestine (B) and brain (C) tissues in the control group

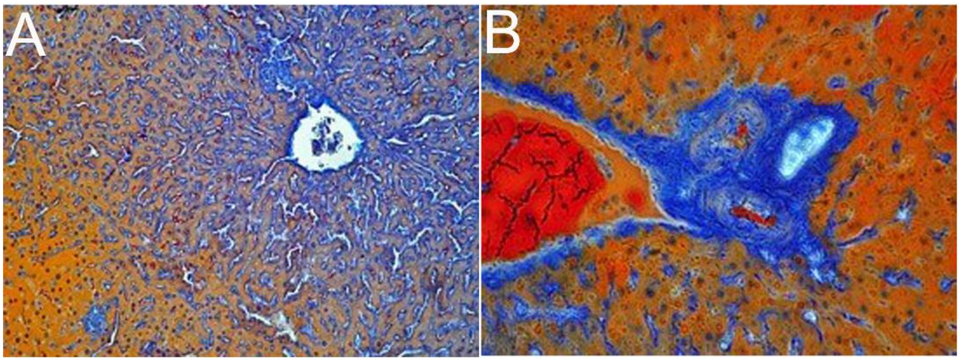

Figure S2 Mallory's Trichrome Staining of liver tissues. There is hepatic sinusoidal reticular fiber turn into collagen in liver (A, 20×) and proliferation of fibrous connective tissue (arrow) in portal tracts (B, 40×).
